# Management Practices, Farmers' Knowledge of Diseased Fish, and Their Occurrence in Fish Farms in Nyeri County, Kenya

**DOI:** 10.1155/2021/8896604

**Published:** 2021-02-18

**Authors:** Isaac R. Mulei, Paul G. Mbuthia, Robert M. Waruiru, Phillip N. Nyaga, Stephen Mutoloki, Øystein Evensen

**Affiliations:** ^1^Norwegian University of Life Sciences, Faculty of Veterinary Medicine, P.O. Box 369, Oslo 0102, Norway; ^2^Department of Veterinary Pathology, Microbiology & Parasitology, Faculty of Veterinary Medicine, University of Nairobi, P.O. Box 29053-00625, Nairobi, Kenya

## Abstract

In this study, fish farmers' management practices, occurrence, and knowledge of fish diseases in Nyeri County, Kenya, were evaluated. Fish farming management practices for small-scale farmers in Kenya have numerous challenges which have led to disease occurrence and reduced production. Moreover, the impact and association of these challenges to farmers' knowledge of fish diseases and their burden has not been fully studied. A semistructured questionnaire was used to capture farmers' biodata, fish species farmed, and farmers' management practices such as handling of nets, pond fertilization, and disposal of fish waste. Farmers' knowledge of fish diseases was based on their ability to identify independent and dependent variable indicators. Independent variables included clinical signs, decreased feeding, bulging eyes, floating on water, abdominal swelling, bulging eyes, abnormal skin color, reduced growth, and abnormal swimming with fish death as were the dependent variable. A total of 208 farmers were interviewed and included those of tilapia (134), mixed tilapia and catfish (40), catfish (22), rainbow trout, and five dams under cooperative management. Tilapia was the most kept fish species (66.8%) followed by polyculture of tilapia and catfish (20%) and rainbow trout (2%). Most respondents were male (78.5%) over 51 years of age (50%). Fifty percent of the respondents had secondary school education. There was a significant association between deaths and sharing of nets in Kieni East subcounty (*p*=0.0049, chi-square), while on-farm fish waste disposing appeared to cause higher deaths compared to burning of the waste although not statistically significant (*p*=0.13). Few respondents observed decreased feed uptake (<20%) and poor growth. Fifty-seven percent of farmers reported mortalities. Fish poor growth, floating in water, and management practices in subcounties had significant effect on fish deaths. The farmers had knowledge of signs of diseased fish, but there was paucity of knowing the specific causes of disease. Farmers need to be empowered on best aquaculture husbandry to avoid disease transmission and specific fish disease signs to enhance proper reporting of disease for subsequent mitigation measures.

## 1. Introduction

The Economic Stimulus Programme (ESP) of 2009 brought with it increased fish farming in Kenya, and at the same time, new challenges unfolded including limited knowledge of aquaculture management practices, nutritional requirements for optimal fish productivity and biosecurity [1], and occurrence and spread of fish diseases [2, 3]. In Nyeri County, fish farming is both commercial and subsistence with many farmers primarily practicing subsistence farming for family consumption. This has led to improved family food security and improved income for commercial farmers [4]. It has been demonstrated that successful fish farming besides understanding the fish biology requires knowledge and expertise of water quality management, proper nutrition, and understanding occurrence and causes of disease, summarized as aquaculture management skills [5]. Indeed, diseases and accompanying poor health reported in Nile tilapia and African catfish hatcheries were associated with poor aquaculture management practices [6]. A similar trend was also observed in rural parts of Côte d' Ivoire where aquaculture failed because the farmers lacked adequate aquaculture management skills [7].

In Kenya, fish farming management practices for small-scale farmers have many challenges [8] that have led to poor productivity and disease occurrence. Some of these challenges include practices like sharing of nets, poor disposal of fish waste, and uncoordinated promotion of fish farming [9]. Furthermore, there is inadequate farmers' and extension workers' training, extension services, certified quality fingerlings, and affordable commercial feeds. In addition, insufficient legislations and policies on fish farming have also led to poor management, reduced research effectiveness, and reduced investment [9]. This has led to constrained production and growth to small-scale farmers compared to commercial farmers [4]. The impact and associations of these challenges to farmers' practices, knowledge of fish diseases, and fish disease burden has not fully been documented in Kenya. Therefore, the aim of this study was to evaluate fish farmers' management practices, their knowledge of fish diseases, and potential association between practices and occurrence of fish diseases in Nyeri County. This would be done with a view of coming up with solutions like establishing diagnostic protocols and communication channels including training workshops, policy briefs, and ultimately build capacity in fish health.

## 2. Materials and Methods

### 2.1. Study Area

**Figure 1:**
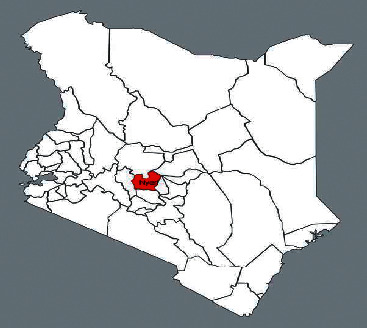
Map of Kenya showing Nyeri County (red). Map courtesy of Maphill.

**Figure 2:**
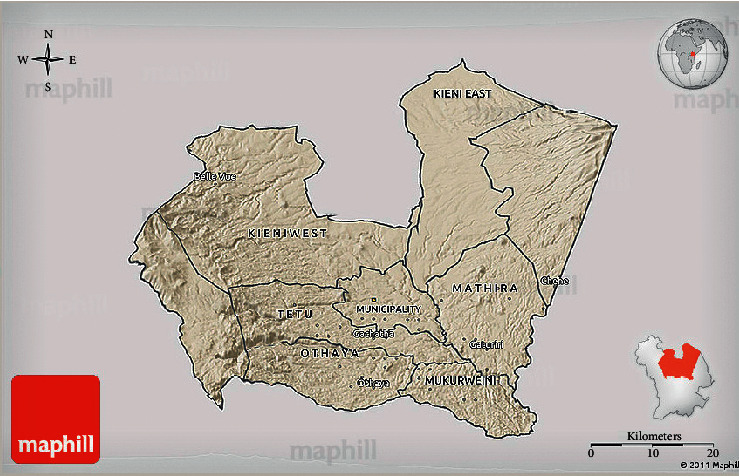
Map of Nyeri County showing the different subcounties (municipality represents Nyeri Central). Map courtesy of Maphill.

Nyeri County is situated between longitudes 36°038′East and 37°020′East and latitude 00° 380′South (Figures 1 and 2) with temperature ranges between 12°C and 27°C and annual rainfall averages of 550 mm in lowland and 1500 mm in the highlands. Altitudes of study subcounties ranged from 2130 to 2380 m (Tetu), 2070 to 2400 m (Kieni East and West), and 1830 m (Nyeri Central and Othaya) above sea level. The county is prone to both drought and floods since some areas are semiarid (those lying on the leeward side of Mt. Kenya) and others very wet. Apart from fish farming, the area is suitable for agriculture with farmers growing cash crops, for instance, tea and coffee and subsistence crops such as maize, beans, and bananas. Dairy, beef, poultry, and pig farming are also practiced [10].

### 2.2. Study Design and Sampling

This study was carried out as a cross-sectional study in five subcounties of Nyeri County between 24^th^ and 29^th^ of August 2015. The questionnaire was organized as a semistructured questionnaire, and after being prepared, it was pretested on a selected number of farmers; a final version that incorporated the results from the pretest was prepared and used for all subsequent interviews. The information gathered from the fish farmers was done through a face-to-face interview by the researcher with the help of enumerators (12 persons in total involved).

The interviews were done with respondents who were either the owners of the fish farms (*n* = 42), farm managers (*n* = 21), caretaker (*n* = 72), or pond attendant (*n* = 17) and the remaining having combined responsibilities (owner/caretaker *n* = 12) or serving different functions in the local society (*n* = 42). The questionnaire focused on fish farm details including type of pond (earthen, earthen with linen, wooden-linen, and concrete), source of water (flowing or stagnant), and age and species of fish and reported mortalities, management practices including pond fertilization, pond drainage and treatment, pond water testing, source of fingerlings, sharing of fingerlings, sharing of water sources, sharing and treatment of nets, and disposal of fish waste. For fish health, we focused on indicators of clinical signs of disease, i.e., feed uptake, poor growth, stationary fish, abnormal swimming behavior, skin lesions, abnormal skin color, swollen abdomen, and floating of fish in the ponds and scored as absent (0) or present (1). Farm management practices included ownership and usage of net, methods of net cleaning, fishpond types, sources of water, flow of water, pond fertilization, and disposal of fish waste after processing. The above parameters were chosen to provide information about disease occurrence and association with management practices. The interviews were combined with on-farm visits to the farms.

The subcounties studied were Kieni East, Kieni West, Nyeri Central, Othaya, and Tetu. A list of fish farmers who owned at least one pond was obtained from the Director of Fisheries in the county, and this list formed the sampling frame. Samples were purposively drawn from all the subcounties. In this study, households were the sampling units used. For each subcounty the following farmers were interviewed: Kieni East (*n* = 51), Kieni West (*n* = 30), Nyeri Central (*n* = 37), Othaya (*n* = 41), and Tetu (*n* = 49).

### 2.3. Data Analysis

Data were entered in Microsoft office Excel (2016) and exported to Stata15 for descriptive and inferential statistics. The chi-square test of independence was used to determine if there was a significant association between two categorical variables. An alpha level of 0.05 was used for all statistical tests.

## 3. Results

### 3.1. Farming of Fish Based on Gender

**Figure 3:**
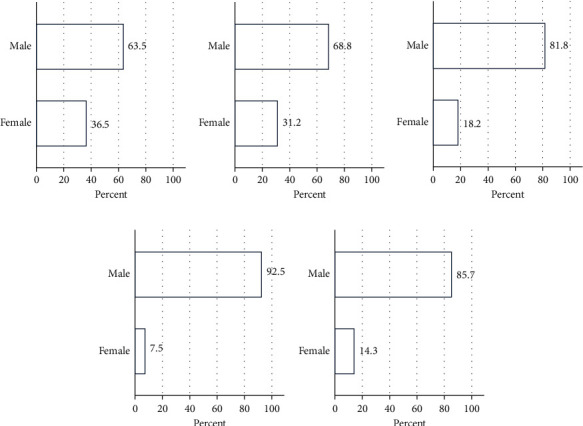
Percentage of male and female respondents in the five subcounties. There was male domination of fish farming in all the subcounties, with high disparity observed in Othaya subcounty. High female participation was observed in Kieni East subcounty. (a) Kieni East. (b) Kieni West. (c) Nyeri central. (d) Othaya. (e) Tetu.

Generally, there were more male than female farmers among respondents. Tetu subcounty had the highest percent male respondents, while Kieni West had the fewest. Kieni East had the highest percentage of female farmers, while Othaya had the least (Figure 3).

### 3.2. Fish Farming Based on Fish Species

**Figure 4:**
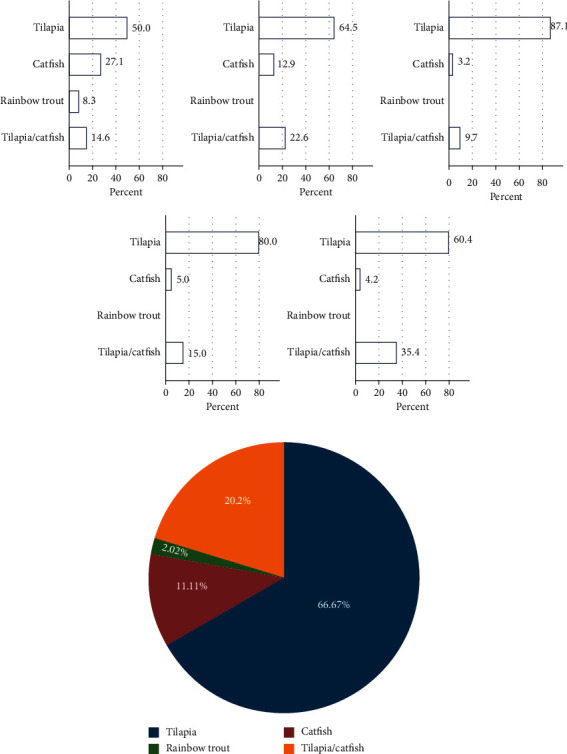
(a) Percentage of fish species farmed per subcounty. Tilapia was the most farmed species in all the subconties, while rainbow trout was only farmed in Kieni East subcounty. (b) Distribution for all subcounties is shown in Figure 4(b).

Overall, the most farmed fish species was tilapia, farmed in 66.8% of the farms with the highest in Othaya and Tetu (Figures 4(a) and 4(b)), while catfish as a single species came second, farmed in 11.1% of the farms. Rainbow trout was the least farmed at 2%. A polyculture of catfish and tilapia accounted for 20.1% of the farms (Figures 4(a) and 4(b). Consistent with gender demographics of respondents (Figure 3), male farmers dominated fish farming in general.

### 3.3. Age and Education of Respondents

**Figure 5:**
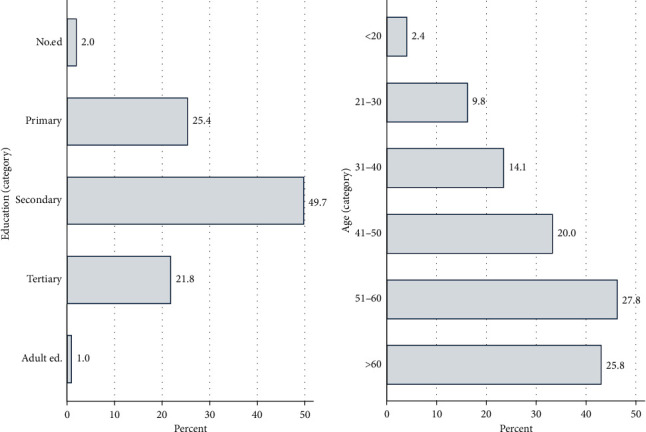
Education level and age distribution among respondents. More than 70% of respondents have secondary/tertiary education and more than 70% are 40 years and older. (a) Education distribution-respondents. (b) Age distribution-respondents.

The age distribution and level of education for the respondents are shown in Figure 5. Few respondents were below 20 years of age. For all subcounties except for Kieni East, 50% of respondents were aged 50 years and above (Figure 5). The level of education was secondary level and below for >75% of respondents (Figure 5). Kieni East was the only subcounty that had a respondent with adult education (Figure 5). Education levels varied between subcounties with Nyeri Central and Tetu being the subcounties with the highest proportion of secondary education and higher (Supplementary Figure 1).

### 3.4. Management Practices

#### 3.4.1. Ownership and Usage of Fishing Nets

Usage of fishing nets for harvesting fish in the subcounties was a common practice, and these nets were shared among farmers in Nyeri County, with 23.5% of farmers sharing in Kieni East, 21% in Othaya, and 10% in Nyeri Central. Reported fish deaths were high in Kieni East and Othaya, where the highest percentage of shared nets was reported, but not statistically significant difference (*p* > 0.05 chi-square).

#### 3.4.2. Treatment of Nets Postharvesting

**Figure 6:**
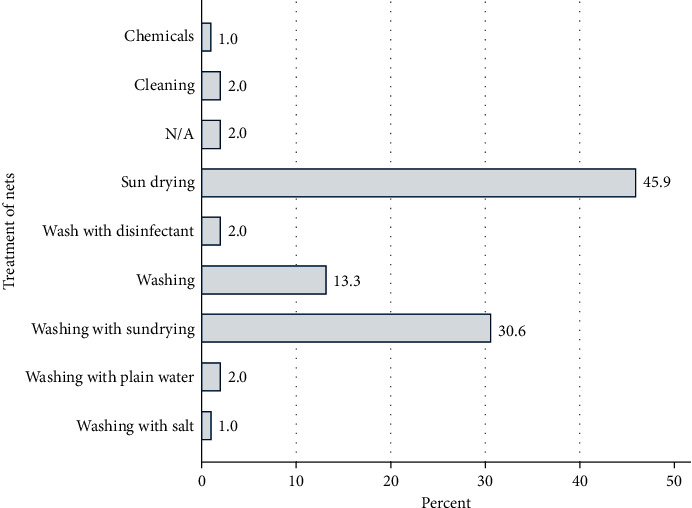
Different treatments of fishing nets postharvest in Nyeri County. Most of the farmers used washing and sun drying for treatment of nets, with very few using chemicals or salt.

**Figure 7:**
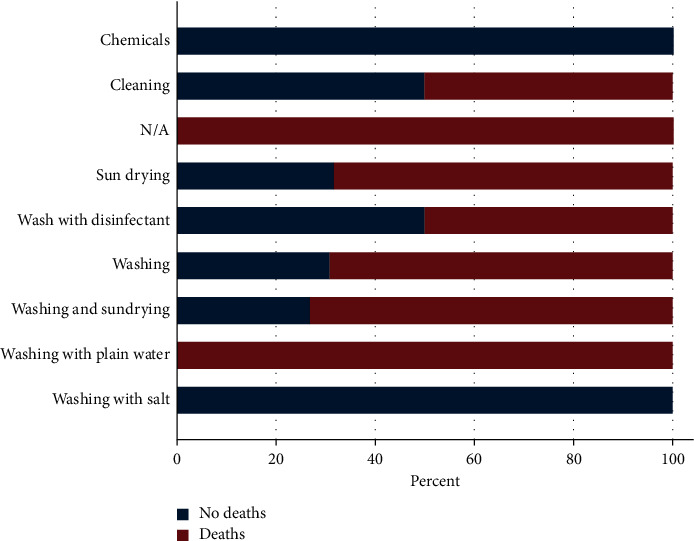
Deaths (percentage, *x*-axis) reported in farms using different methods of net disinfection after harvesting. Most deaths (100%) were recorded in farms using plain water to clean nets, while there were no deaths when chemicals and salt were used.

Regarding treatment of nets after fish harvesting, 45.9% and 30.6% of the respondents' sun-dried or combined sun drying with washing of the nets, respectively (Figure 6). Very few respondents used chemicals (1.0%), salt (1.0%), disinfectants (2.0%), or simple washing with water (2%) (Figure 5). Sun drying only was the dominating practice in Kieni East (80%) and West (50%) and Othaya (73.3%), while Nyeri Central (83.3%) and Tetu (44.7%) used a combination of washing and sun drying. Occurrence of deaths in ponds was grouped according to postharvest treatments of nets (Figure 7), and when “chemical” and “washing with salts” treatments were used, no deaths were reported. The total number of sites reporting these treatments was low (*n* = 2), and hence, interpretation of results should be made with caution. Comparison of washing and washing/sun drying did not give significant difference in deaths (*p* > 0.05, chi-square), and the same holds for any kind of washing (combined with disinfectant or sun drying) versus sun drying only (*p* > 0.05).

#### 3.4.3. Pond Types

Farmers in the county reared fish in four types of ponds: earthen, earthen-linen, wooden-linen, and concrete ponds. Tilapia was mainly farmed in earthen and earthen-linen ponds (95%), catfish was reared in mixed ponds, rainbow trout in concrete ponds, and tilapia/catfish combinations in earthen or earthen/linen environment (Figure 7). For tilapia, deaths reported for the different production environments were proportional to the number of each environment, and for catfish, the same was found. For tilapia/catfish combined, deaths were more frequently reported in earthen-lined ponds compared to earthen ponds; i.e., 62.5% of reported deaths were in earthen-linen ponds, while they constituted only 46.2% of the environment used (Figure 7) and 33.3% of deaths were in earthen ponds (*p*=0.006).

#### 3.4.4. Sharing of Water Source

**Figure 8:**
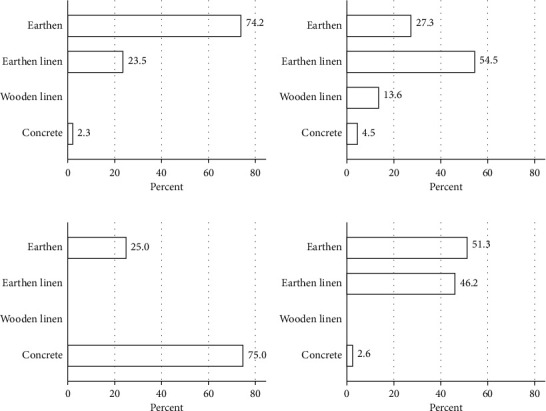
The type of pond used for farming each fish species. Tilapia was mainly reared in earthen ponds and catfish in earthen-linen, while rainbow trout was reared in concrete ponds. (a) Tilapia. (b) Catfish. (c) Rainbow trout. (d) Tilapia/catfish.

Ponds in the county were mainly supplied by river water, either free flowing or piped (Figure 8Q1: There was no in-text citation for Figure 8. Please check the suggested in-text citation given for Figure 8 and correct its placement if necessary.). Water source was shared for fish farms especially in Kieni East and Kieni West. There was no significant association between sharing water source and deaths of fish.

#### 3.4.5. Flow of Water

In fishponds, water was either flowing or stagnant. In farms rearing tilapia and catfish, the pond water was mainly stagnant (>90% of ponds). Trout farming was mainly practiced in flowing water. There was slight increase in deaths recorded in stagnant water in tilapia and catfish and however not significantly different (*p* > 0.05).

#### 3.4.6. Fertilization of Ponds

**Figure 9:**
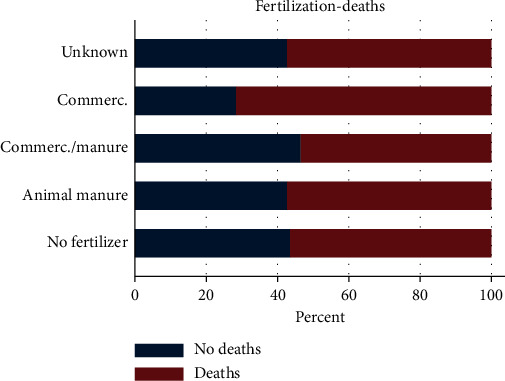
Deaths recorded for types of fertilization, all species and production systems combined. More deaths were recorded in farms using commercial fertilizers.

Most respondents (>60%) used animal manure to fertilize ponds but with variations between the species farmed. Ponds that used commercial fertilizers reported more fish deaths compared to those using organic manure and were however not significantly higher (Figure 9).

#### 3.4.7. Disposal of Fish Waste after On-Farm Slaughtering

The practice of slaughtering fish on-farm was quite common, and methods of disposing fish waste were determined. Majority of the respondents, 39.7% and 33.3%, disposed their fish waste by throwing back into the pond(s) and giving to other animals in the farm, respectively. Very few respondents (0.6%) reported burning their waste. When “disposing on farm” was compared to “bury/remove,” the reported deaths were higher, however not statistically significantly different (*p*=0.13, chi-square).

### 3.5. Clinical Signs of Disease Observed by Farmers

**Figure 10:**
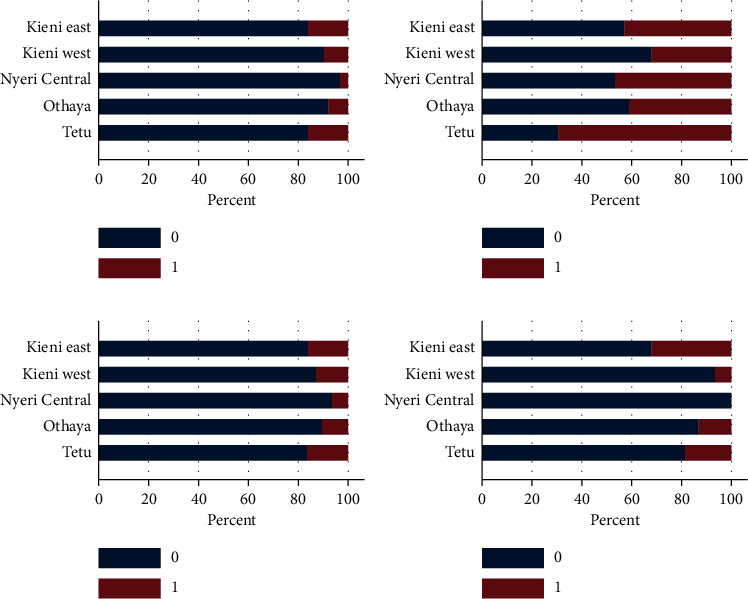
Clinical signs as observed by farmers in different subcounties. 0 = not observed; 1 = observed. Percentages are per clinical sign/observation. Most respondents were able to identify poor growth as a clinical sign of disease. (a) Decreased feed. (b) Poor growth. (c) Stationary. (d) Abnormal swimming.

**Figure 11:**
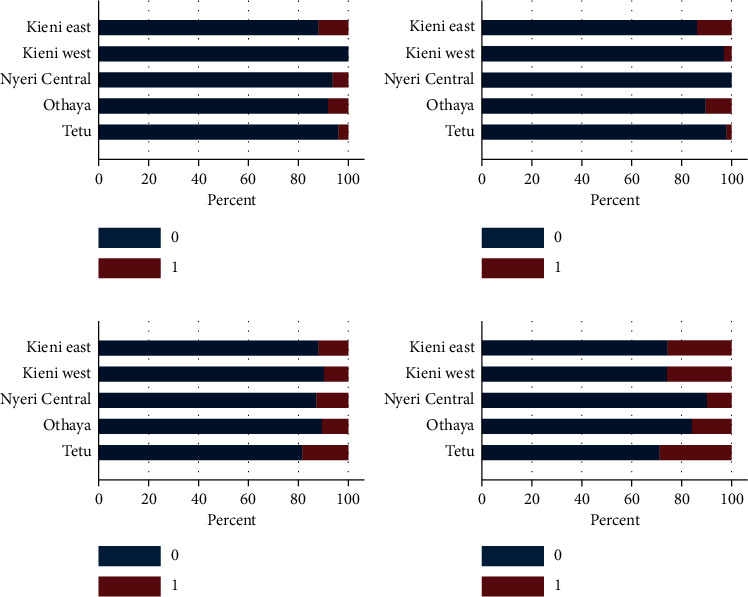
Clinical signs as observed by farmers in different subcounties. 0 = not observed; 1 = observed. Percentages are per clinical sign/observation. Higher percentage of farmers identified “floating fish” and “swollen abdomen” as signs of disease. (a) Skin lesions. (b) Abnormal skin color. (c) Swollen abdomen. (d) Floating fish.

The scoring per subcounty is summarized in Figures 10 and 11. There was variation between subcounties, while relatively few observed decreased feed uptake (<20%); poor growth was observed in 35% to 70% of the farms, which was highest in Tetu County (Figure 10). Stationary fish and abnormal swimming were observed in less than 20% of the farms, except for Kieni East that observed abnormal swimming at 35% of the farms (Figure 10), and in all farmed species (tilapia, catfish, rainbow trout, and mixed productions). Abnormal skin color was mainly observed in Kieni East (<20%) (Figure 11), while floating fish were seen mainly in Kieni East, Kieni West, and Tetu (>20%) (Figure 11). Over 10% of respondents reported swollen abdomen in all the five subcounties (Figure 11).

### 3.6. Mortalities

**Figure 12:**
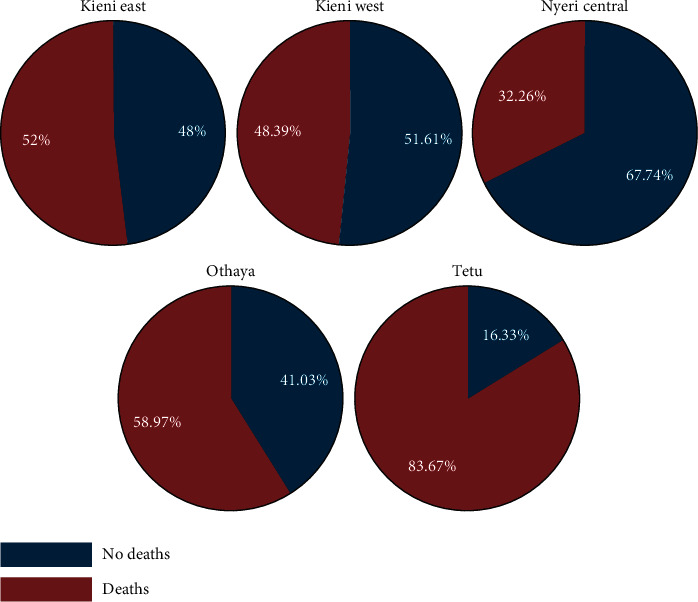
Deaths in different subcounties. Tetu stands out with highest mortality, all species included.

Fifty-seven percent (57%) had observed fish mortalities on their farms, and reported deaths were evenly distributed between farmed fish species. Fish deaths were reported more frequently in Tetu (*p* < 0.01) when compared to all other subcounties (Figure 12). Nyeri Central had the lowest occurrence of mortalities (Figure 12).

**Figure 13:**
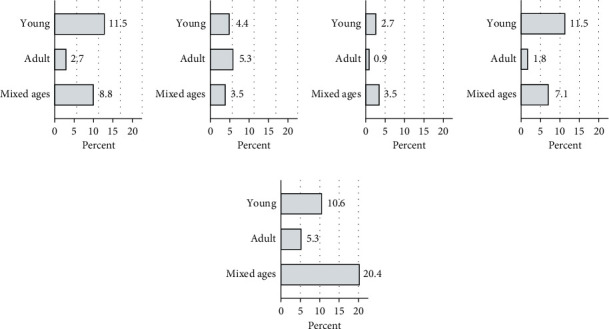
Age distribution of fish reported dead in the different subcounties. There were more deaths recorded in young fish in Kieni East, Othaya, and Tetu subcounties. Kieni West and Tetu recorded higher deaths in adult fish, while Tetu subcounty recorded more deaths in both young and adult fish. (a) Kieni East. (b) Kieni West. (c) Nyeri. (d) Othaya. (e) Tetu.

On average, fish deaths were observed at equal proportions in young fish (41.2%) and mixed age classes (43%), while adult fish accounted for 15.8% of total recorded deaths. For Tetu, the highest proportion of deaths was reported in the mixed ages (Figure 13) and 20.4% of total deaths grouped to this category in Tetu.

Deaths were also recorded by time of year, the occurrences in the different months by subcounty vary, and there were distinct differences with Nyeri Central standing out as having mortalities only during 3 months of the year (April, August, and December), while for Othaya, deaths are spread throughout the year.

A logistic regression analysis for “deaths” (0/1) as the dependent variable and “poor growth,” “fish floating,” and “area” (subcounty) as independent variables included 197 observations and showed an overall statistically significant effect from the model (*p* < 0.0000). Running a marginal analysis, it was shown that the mean predicted probability of death being observed in the ponds based on the observed variables is 0.51, 0.50, 0.37, 0.62, and 0.83 for Kieni East, Kieni West, Nyeri Central, Othaya, and Tetu, respectively.

## 4. Discussion

The main findings from this study are that management practices vary between fish farmers and subcounties and, in some instances, are associated with occurrence of deaths in fish. A high percentage of farmers in the subcounties shared harvesting nets; however, significant association between deaths and sharing of nets was only recorded in Kieni East. Increased deaths were reported in tilapia and catfish reared in concrete ponds (as mono- or polyculture), while for rainbow trout, the highest mortality was observed in earthen ponds (100%). For combined tilapia/catfish farming, deaths were significantly more frequent in earthen compared to earthen/linen ponds. We could not demonstrate that sharing water source, different methods of pond fertilization, on-farm slaughtering, and local disposal of fish waste impacted on occurrence of mortality.

This study evaluated fish farmers' management practices, the occurrence of deaths on farms, and any link between practices and occurrence of deaths. Usage of nets for harvesting fish was a common practice in Nyeri County, and nets were shared between farmers, especially in Kieni East and Othaya. We could link an increased occurrence of deaths with these management practices. We therefore engaged the respondents to find out if these nets were treated after harvesting of fish and the methods employed. Over 45% of the respondents' sun-dried their nets while 30.6% combined sun drying with washing. Very few respondents used chemicals (1.0%), salt (1.0%), and disinfectants (2.0%). The study established that more deaths were recorded in farms that used washing for treatment of nets compared to those using chemicals and salts. Indeed, it was documented [11] that one of the important routes by which pathogens could be introduced into and spread between fish populations in different water bodies was through contaminated nets.

The study showed that, in Nyeri County, tilapia was the most farmed species (66.8%) followed by African catfish (11.1%) and rainbow trout (2%). This agrees with a previous study [12] which showed that, under the ESP of 2009, 96.1% and 94.6% of fish farmers in the counties of Kiambu and Machakos adopted tilapia monoculture. However, the picture is different worldwide, where tilapias are the second most farmed fish after carps [13].

Ponds in the county were mainly supplied by river water, either free flowing or piped. Therefore, many ponds were sharing water source especially in Kieni East and Kieni West (>70%). While there was no significant association between sharing water source and deaths of fish in our study, this practice has been shown to aid in introduction and distribution of pathogens in fish farms through horizontal transmission [14, 15].

It was established that the common methods of pond fertilization were through use of animal manure, commercial fertilizers, and a combination of both. Most of the respondents (>60%) used animal manure to fertilize their ponds compared to (>7%) that used commercial fertilizers. A similar trend has been documented [1, 16]. However, in both these studies, the association between pond fertilization and fish mortalities was not studied, and in one of them [16], emphasis was on tilapia and catfish. Most fish farmers practicing pond culture use manure or inorganic fertilizer in ponds to increase the supply of natural food organisms for fish so as to reduce production costs arising from commercial feeds [17]. The manures in use are mainly cow dung, sheep, poultry, and rabbit manure. While addition of these manures has been shown to increase the risk of introduction of pathogens into the system [18], in our study, deaths were equally reported in ponds that were not fertilized.

Most farmers slaughtered their fish on farm. It was determined that, after slaughtering, farmers mainly disposed the waste by throwing it back into the ponds (39.7%) or giving as feed to other animals (33.3%). Previous studies have shown that poor disposal of fish waste could lead to transmission and distribution of pathogens [19]. The possibility of recirculating pathogens through such practices is obvious, and better principles of disposal should be instituted.

From the study, it was observed that farmers' literacy levels were good with only 2% of the respondents having no formal education. Majority of the respondents (40.7%) had secondary education with 21.8% acquiring tertiary education. We suggest that education would be important in fish farming because it avails knowledge that is key to understanding indicators of diseases. In terms of fish farming, most of the respondents were >40 years old. This could be attributed to long time experience of the older generation in fish farming either through training or exposure and the subsequent benefits accrued from fish farming.

A healthy fish has its natural schooling behavior in the water. A deviation from this may indicate a health problem. In this study, we asked farmers if they had observed any abnormal behavior of fish in terms of swimming and general activity in the water. Stationary fish and abnormal swimming were observed by less than 20% of the farms, except for Kieni East that reported abnormal swimming at about 35% of the farms. It is important to note that most of the farms in Kieni East were rearing rainbow trout. More than 20% of respondents observed fish floating on water surface. Fish are usually active, and therefore, change in behavior such as color, floating, sinking, and hiding is often signs of a sickness. From the results, it is evident that considerable numbers of respondents were able to identify behavior of fish that were associated with deaths. In fact, from the study, we have demonstrated a significant association between fish death and abnormal swimming and floating on water surface, especially in rainbow trout fry. This finding is as reported elsewhere [20, 21] and has been associated with insufficient knowledge and awareness of fish farmers.

It was observed that respondents, although low in number, were able to identify clinical signs of disease that were associated with deaths of fish. Less than 20% of respondents observed decreased feed intake, while 35–70% observed poor growth. There was a significant association between poor growth and fish deaths. Such a challenge has been reported previously [22], where 23% of farmers said that they could identify some diseases, while 15.6% could not recognize any disease. In addition, the state of appetite is important as it is indicative of the status of health of fish. Anorexia in fish has been associated with an underlying disease [23] and poor growth leading to retardation [24, 25]. However, it was observed that farmers were keen to note any deaths in their ponds with 57% of respondents reporting fish deaths in their ponds. The reported deaths were evenly distributed between fish species farmed. Fish deaths were reported at a higher percentage in Tetu followed by Othaya and Kieni East. Nyeri Central had the lowest percentage of reported mortalities.

The study has shown that Tetu subcounty recorded the highest risk in fish morbidity and mortality with the highest proportion of deaths reported in both young and adults. In fact, a chi-square test showed a significant association between death and areas where the fish were farmed. We suggest that this could be due to farming the wrong species in the subcounty. The subcounty lies at an altitude of over 2000 meters above sea level with water temperatures measuring as low as 9°C which is not conducive for tilapia farming, yet this species was being farmed in the region. The optimal growing temperatures for Nile tilapia are between 22°C and 29°C; these fish may be unable to survive at temperatures below 10°C, and growth is poor below 20°C [26, 27]. This is unlike the Blue tilapia (*Oreochromis aureus*) which is tolerant to cold climate [28]. In addition, significant association of clinical signs observed, and death was shown in Kieni East. Many of the rainbow trout farms were located in this subcounty, and therefore, it was likely that recorded importation of breeding material may have introduced pathogens to the rainbow trout farms [29].

There were more male than female farmers who responded to the questionnaire. Tetu subcounty had the most male respondents, while Kieni West had the fewest. Kieni East had the most female respondents, while Othaya had the least female respondents. While there was no significant association between fish species farmed and gender of the respondents, there was a significant association between gender of respondents and the subcounty. This could be attributed to land ownership where men own land and tend to be the primary investors, while women are involved later during fish processing. This finding agrees with other studies [30]. Another study [31] also reported forms of gender inequalities within the aquaculture sector in Trans Nzoia County, Kenya. Furthermore, another study [32] showed that women constitute more than one-fourth of people working in fisheries and aquaculture sector. In Zambia, it was reported that while women were involved in small-scale fish farming and in livestock husbandry, their contribution to fisheries was minimal compared to men [33]. However, a study in Tanzania showed an increased participation of women in aquaculture [34] especially around Lake Victoria with up to over 60% participation.

From this study, we conclude that farmers' management practices are likely to contribute to introduction and distribution of fish pathogens in Nyeri County. Secondly, farmers have knowledge on clinical signs of diseases in fish but could not identify the specific diseases. Therefore, on the basis of the information collected, there is need for follow-up training of fish farmers and other stakeholders on proper management practices and implementation of good biosecurity principles to prevent and control introduction and transmission of fish pathogens in the farms. Results of this study should also be shared with the relevant government authorities for proper implementation through establishment of extension services and capacity building. Follow-up studies to these findings have already motivated investigations into likely causes of fish mortalities in the county, and more research is required to determine the presence and prevalence of diseases causing such deaths especially in rainbow trout fry. In addition, creation of linkages and collaboration between research institutions, universities, fish farmers, nongovernmental organizations, fisheries officers, and policymakers are needed so as to facilitate dissemination of information on best fish farming practices and disease occurrence and control.

## Data Availability

The data are stored in cloud-based programs. The data used to support the findings of this study are available upon request.

## Supplementary Materials

Supplementary Materials Supplementary Figure 1. Here, we have analyzed the outcome death based on which county the fish were farmed (subcounty differentiation). The contrast analysis after margins analysis for prediction of death of fish in the different subcounties, using Nyeri Central with the lowest recorded mortality as baseline, shows that Othaya and Tetu have significantly higher prediction of deaths. This can be seen from the margin plot below, where confidence limits do not cross the 0-line for Othaya and Tetu (vs. Nyeri Central). Supplementary Figure 2. Here, the margin analyses are performed to compare each county against average deaths for all counties. Nyeri Central has significantly lower prediction for deaths than average, and Tetu is significantly higher, as can be seen from the table and the margin plot (where confidence limits do not cross the 0-line for Othaya and Tetu (vs. mean).
