# Supplementary material for: Management Practices, Farmers' Knowledge of Diseased Fish, and Their Occurrence in Fish Farms in Nyeri County, Kenya
Source: Vet Med Int. 2021 Feb 18;2021:8896604. doi: 10.1155/2021/8896604 (PMC7910046; doi:10.1155/2021/8896604)

```
Contrasts of adjusted predictions
Model VCE    : OIM

Expression   : Pr(deaths), predict()
```

| | df | chi2 | P>chi2 |
|---|---|---|---|
| d3 | | | |
| (Kieni East vs Nyeri Central) | **1** | **3.24** | **0.0720** |
| (Kieni West vs Nyeri Central) | **1** | **1.72** | **0.1894** |
| (Othaya vs Nyeri Central) | **1** | **5.39** | **0.0203** |
| (Tetu vs Nyeri Central) | **1** | **26.87** | **0.0000** |
| Joint | **4** | **33.81** | **0.0000** |

Supplementary Figure 1. Here we have analyzed the outcome *death* based on which county the fish were farmed (subcounty differentiation). The contrast analysis after margins analysis for prediction of *death* of fish in the different sub-counties, using Nyeri Central with the lowest recorded mortality as baseline, shows that Othaya and Tetu have significantly higher prediction of deaths. This can be seen from the marginsplot below, where confidence limits do not cross the 0-line for Othaya and Tetu (vs. Nyeri Central).

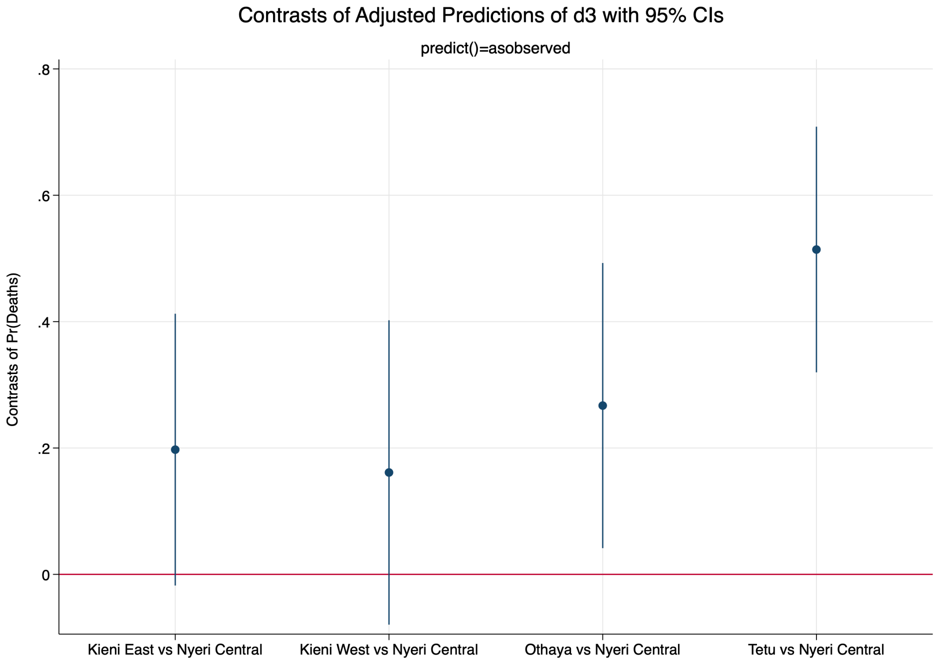

Supplementary Fig. 2. Here the margins analysis is performed to compare each county against average *deaths* for all counties. Nyeri Central has significantly lower prediction for deaths than average, and Tetu significantly higher, as can be seen from the table and the the marginsplot (where confidence limits do not cross the 0-line for Othaya and Tetu (vs. mean).

Contrasts of adjusted predictions
Model VCE : **OIM**

Expression : **Pr(deaths), predict()**

| | df | chi2 | P>chi2 |
|---|---|---|---|
| d3 | | | |
| (Nyeri Central vs mean) | **1** | **9.64** | **0.0019** |
| (Kieni East vs mean) | **1** | **0.22** | **0.6353** |
| (Kieni West vs mean) | **1** | **0.74** | **0.3890** |
| (Othaya vs mean) | **1** | **0.31** | **0.5753** |
| (Tetu vs mean) | **1** | **28.87** | **0.0000** |
| Joint | **4** | **33.81** | **0.0000** |

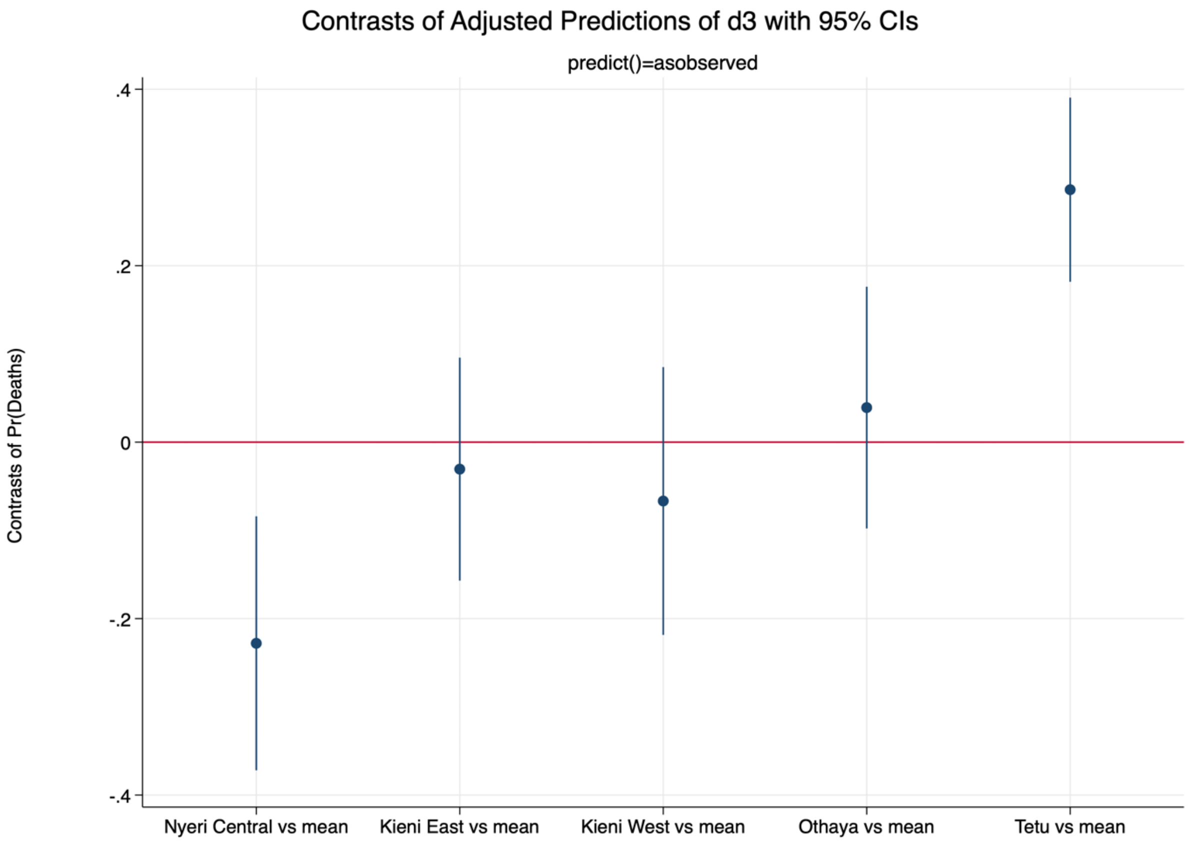

Supplement: Supplementary Materials — Supplementary Figure 1. Here, we have analyzed the outcome death based on which county the fish were farmed (subcounty differentiation). The contrast analysis after margins analysis for prediction of death of fish in the different subcounties, using Nyeri Central with the lowest recorded mortality as baseline, shows that Othaya and Tetu have significantly higher prediction of deaths. This can be seen from the margin plot below, where confidence limits do not cross the 0-line for Othaya and Tetu (vs. Nyeri Central). Supplementary Figure 2. Here, the margin analyses are performed to compare each county against average deaths for all counties. Nyeri Central has significantly lower prediction for deaths than average, and Tetu is significantly higher, as can be seen from the table and the margin plot (where confidence limits do not cross the 0-line for Othaya and Tetu (vs. mean). [file 8896604.f1.pdf]
